# Influence of biopsychosocial factors on a functionally delayed ageing process

**DOI:** 10.1007/s00404-024-07885-5

**Published:** 2024-12-24

**Authors:** Susanne Theis, Norman Bitterlich, Mirjam Moser, Michael von Wolff, Petra Stute

**Affiliations:** 1 Department of Obstetrics and Gynecology, University Medical Center of Johannes Gutenberg University Mainz, Mainz, Germany; 2 Draisdorfer Str. 21, 09114 Chemnitz, Germany; 3 Department of Obstetrics and Gynecology, Inselspital, Bern, Switzerland

**Keywords:** Bio-functional age, Bio-functional status, Active and healthy ageing, Influenceable factors

## Abstract

**Purpose:**

Increasing life expectancy and rising populations create new challenges for science, economy, politics, society and each individual. The bio-functional status (BFS) as a theoretical model incorporates the International Classification of Functioning (ICF) and the concept of active and healthy ageing (AHA). This study addresses the question of which the strengths and resources have the greatest positive impact on bio-functional age (BFA) and might be influencable.

**Methods:**

A monocenter, cross-sectional, observational, non-interventional trial was performed from 2012 to 2014 at Inselspital Bern to evaluate the BFS, a complex, generic, non-invasive, sex- and age-validated assessment tool. A standardized battery of assessments was performed on 464 females and 166 males, aged 18 to 65 years (*n* = 630). We aimed to statistically identify BFS items that might be influenceable to support healthy ageing and vitality.

**Results:**

341 participants of the original cohort were included. After carrying out regression analysis, 10 parameters (*T* = 8.992; *p* < 0.001) remained as possible variables that can be influenced (*R*^2^ = 0.758). Of those identified parameters, one can be assigned to subcategory I of BFS (pulse performance index), two to II (tapping frequency part I and II), two to III (strategy building and verbal reaction time) and three to IV [sense of coherence, social potency, complaint questionnaire (BFB total)]. Age and sex, nevertheless, have an influence on the BFA and the BFA-Index.

**Conclusion:**

The most promising approach to support vitality, is to support low social stress, high social integration, a good sense of coherence and maintaining a good mental and cognitive status.

**Supplementary Information:**

The online version contains supplementary material available at 10.1007/s00404-024-07885-5.

## What does this study add to the clinical work

**Table:** 

We therefore believe that the application of BFS at the individual level can help people to achieve greater vitality and thus quality of life as they grow older. At the same time, this could relieve the burden on societies, politics and economies and thus make an important contribution to the global project of the active and healthy ageing.	

## Introduction

Life expectancy and population are increasing all over the world. In 2060 10.1 billion people are expected to live worldwide (for comparison in 2024, 8.2 billion people were counted) [1]. According to data from the World Health Organization (WHO), life expectancy at birth in Europe in 2000 was 72.5 years, has increased lately to 78.2 years and is expected to raise up to 83.9 years in 2060. In the European Union the share of people aged 65 to 79 years and older has increased from 12.6% in 2003 to 15.3% in 2023 as well as the proportion of people 80 years and older from 3.7% in 2003 up to 6.0% in 2023 [2].

Increasing life expectancy and rising population figures can on the one hand be seen as a success and result of better living conditions and advances in medicine. On the other hand, this creates new tasks and challenges for science, economy, politics and society, as well as for each individual. Physical and mental health as well as social integration, self-determination and economic prosperity into old age should be maintained. From an economic point of view, individuals ageing in health create more added value than those ageing in illness. They are no longer employable in the labor market and create less costs for state systems, as they are less dependent on support services, such as pensions or health insurance benefits. On an individual level, every additional year of life spent in physical and mental health means a better quality of life. Thus, addressing the problem of an ageing population is one of the main priorities of WHO [3].

Healthy ageing is defined as “the process of developing and maintaining the functional ability that enables well-being in older age” by WHO [3]. The concept of active and healthy ageing (AHA) enables people to lead a health-promoting lifestyle and to participate in economic and social life on an ongoing basis aiming for a high degree of self-determination and independence at an older age [4, 5]. AHA incorporates biological ageing, active ageing, as well as changes in psychological and social well-being that may support survival to old age, delay in the onset of chronic non-communicable diseases (NCD), and optimal functioning for the maximal time period at individual levels, body systems, and cells [6]. The corresponding conceptual AHA framework [6] includes several items such as functioning (individual capability and underlying body systems), wellbeing, activities and participation, and diseases, including NCD. As such, AHA is closely related to bio-functional ageing.

The bio-functional status (BFS) assessment tool has been fitted into a theoretical model incorporating both, the International Classification of Functioning (ICF) and AHA concept. This complex, generic, non-invasive, sex- and age-validated assessment tool meets the European Innovation Partnership on Active and Healthy Ageing (EIP–AHA) requirements for a diagnostic AHA instrument. Their requirements comprise applicability to health and disease across age stages (non-pediatric and non-geriatric lifetime), easy, partly self- and proxy administration, and accordance with the ICF of the World Health Organization (WHO).

The present study addresses the question of which the strengths and resources have the greatest positive influence on the bio-functional age and therefore might be influencable. The bio-psychosocial or bio-functional age describes the functionality of a person in the physical, cognitive-mental and emotional-social areas. The best possible physical, cognitive-mental and emotional-social state for a person to strive for in the sense of active ageing is extremely individual and depends only to a small extent on chronological age. For this study, the measures of biopsychosocial age were the Bio-functional Status (BFS) and Bio-functional Age (BFA) assessment according to Pöthig et al. [7]. The BFS assessment tool covers a physical, mental-cognitive, emotional and social domain. Using the BFS, differences on a biopsychosocial level between functionally delayed, normal or prematurely ageing subjects were identified in a study population of 341 subjects from the Bern Cohort Study 2014 (BeCS-14). Thus, factors could be identified that are essential for a delayed functional ageing process and the maintenance of vitality and potentially may be influenced. At best, these findings can be used for future research on healthy ageing to enable as many people as possible to age healthily at an individual level, but also to counter the growing and ageing population at a societal level.

## Material and methods

### Study population

Between 15.05.2012 and 04.07.2014, 464 German-speaking women and 166 men aged 18 to 65 were recruited at University Women’s Hospital, Inselspital Bern, Switzerland. Recruitment was performed by the principle investigator (PS), the study nurse and fourteen doctoral students of the medical school, university of Bern, via personal contact (patients, colleagues, family, friends) and online advertisement (internet, intranet Inselspital Bern, social media). Exclusion criteria were pregnancy, acute diseases (e.g. fever, acute pain syndrome), and illiteracy. The final study cohort included 630 participants, whereof 341 participants (269 female and 72 male participants) were analyzed for the present work since for BFA calculation the age ≥ 35 years is the model-required accepted cut-off value. The study protocol was approved by the Cantonal Ethics Committee Bern (Ref.-Nr. KEK-BE: 023112), and written informed consent was obtained from each participant.

### Study design

The study design of the original study has been described before [8–12]. Briefly, this was a monocenter, cross-sectional, observational, non-interventional trial. All participants (*n* = 630) within BeCS-14 followed a standardized battery of assessments consisting of a personal and family history, bio-functional status (BFS) and derived from it bio-functional age (BFA), as well as validated questionnaires for depression and anxiety (HADS) [13], health-related quality of life (SF- 36) [14] and chronic stress (TICS) [10, 11, 15], respectively. Participants were asked to participate in further assessments addressing “nutrition” by AD-EVA [6, 16] and PATEF [6, 17], “employees’ health” by IMPULS [18], “stress” by heart rate variability [19] and “cognition” by the validated test battery IGD [6, 20]. The assessments and subgroups relevant to this publication are further described in Sect. “Assessment procedures”.

### Assessment procedures

#### Personal and familiy history

Briefly, it was assessed age, social status (partnership, having children, satisfaction with relationship and sex life), lifestyle (alcohol, tobacco, sport, sleep), and job status (highest educational degree, current field of work, job position, working hours, monthly gross income, presenteeism, absenteeism). Personal and family history further comprised information about malignancy, cardiovascular disease, breathing disorder, abdominal and urogenital disease, metabolic disorder, skin and/or hair disease, neuromuscular and psychiatric disorders, as well as bone and joint disease (Table 1a: Comparison of Total Cohort and Subgroups Functionally faster/normal/delayed and Table 1b: Comparison of Total Cohort and Subgroups (metric values).

**Table 1:** (**a**) Comparison of total cohort and subgroups (categorial values). (**b**) Comparison of total cohort and subgroups (metric values)

Parameter	Total cohort (*n* = 341)		Functionally faster (*n* = 85)	Functionally normal (*n* = 168)	Functionally delayed (*n* = 88)	*p* Chi^2^ test (all 3 groups)
	%	95% CI	%	%	%	
(a) Comparison of total cohort and subgroups (categorial values)						
Male	21.1	16.9–25.9	27.1	19.0	19.3	0.098
Female	78.9	74.1–83.1	72.9	81.0	80.7	
Single, living alone, widowed, divorced or other	32.8	27.8–38.2	37.6	32.1	29.5	0.236
Single, but in partnership	11.7	8.5–15.7	15.3	12.5	6.8	0.083
Living in a marriage	55.4	49.9–60.8	47.1	55.4	63.6	0.033
Childless	35.5	30.4–40.9	44.7	36.3	25.0	0.006
University degree	30.8	25.9–36.0	30.6	28.0	36.4	0.182
Advanced technical college	20.5	16.3–25.3	18.8	22.0	19.3	0.549
Vocational training	40.5	35.2–45.9	42.4	41.1	37.5	0.453
Monthly gross income* < 5000 CHF	40.1	34.8–45.6	40.5	39.5	40.9	0.808
Monthly gross income* > 5000 CHF	57.2	51.7–62.6	56.0	58.1	56.8	0.713
No own income*	2.7	1.2–5.0	3.6	2.4	2.3	0.539
Employee status:**						
Executive position	28.5	23.7–33.7	25.0	28.3	32.2	0.337
Employed	66.8	61.4–71.8	66.7	68.7	63.2	0.358
Student	0.6	0.0–2.2	0.0	1.2	0.0	0.303
Jobless	4.2	2.2–6.9	8.3	1.8	4.6	0.012
Job occupation						
> 90%	39.3	34.0–44.8	41.2	41.7	33.0	0.164
50–89%	40.5	35.2–45.9	40.0	41.1	39.8	0.808
< 50%/incl. jobless	19.6	15.5–24.3	18.8	16.1	27.3	0.029
Non smoking	86.2	82.0–89.7	81.2	88.7	86.4	0.108
Physical activity till sweating						
Less than 1 times/week	31.4	26.4–36.6	48.2	26.8	23.9	< 0.001
1–2 times/week	39.9	34.6–45.3	30.6	44.0	40.9	0.031
> 2 times/week	28.7	23.9–33.9	21.2	29.2	35.2	0.054
Alcohol consumption up to 4 times / month	58.7	53.2–64.0	70.6	56.0	52.3	0.008
Alcohol consumption 4 times / week or more	10.9	7.7 – 14.7	7.1	10.1	15.9	0.074
Daily alcohol consumption up to 2 glasses	95.6	92.8 – 97.6	91.8	95.8	98.9	0.020
Satisfaction with partnership quite or very	75.0	70.0 – 79.6	68.2	74.3	83.0	0.021
Satisfaction with sexual life quite or very	57.5	52.0 – 62.9	58.3	52.7	65.9	0.043

(b) Comparison of total cohort and subgroups (metric values)									
Parameter	Total cohort (*n* = 341)			Functionally faster (*n* = 85)		Functionally normal (*n* = 168)		Functionally delayed (*n* = 88)	
	Mean (SD)	95% CI	Median (Q1, Q3)	Mean (SD)	Median (Q1, Q3)	Mean (SD)	Median (Q1, Q3)	Mean (SD)	Median (Q1, Q3)
BMI [kg/m^2^]	25.6 (5.1)	24.7–25.9	24.5 (21.6; 27.4)	26.2 (6.4)	25.2 (21.8; 28.9)	25.1 (4.9)	24.4 (21.7; 27.2)	24.6 (4.0)	24.3 (21.8; 26.6)
Children [*n*]	1.4 (1.3)	1.3–1.6	2.0 (0.0; 2.0)	1.1 (1.2)	1.0 (0.0; 2.0)	1.4 (1.3)	2.0 (0.0; 2.0)	1.7 (1.2)	2.0 (1.2; 3.0)
Duration of sleep [hours]	6.8 (0.8)	6.7–6.9	7.0 (6.0; 7.0)	6.7 (0.8)	7.0 (6.0; 7.0)	6.9 (0.8)	7.0 (6.0; 7.0)	6.8 (0.8)	7.0 (6.0; 7.0)

*CI* Confidence interval of percentage, *n* number, *%* percentage, *CHF* swiss franks

*N = 339

**N = 337

#### Bio-functional status, bio-functional age

The BFS was assessed by a comprehensive test battery developed by Pöthig et al. and reported by others [8, 21–23]. It is used for functionally diagnostic measurement of an individual's vitality. The test battery comprises holistic characteristics from physical, mental-emotional and social areas that fit into a complex theoretical model incorporating the ICF and AHA concept BFS parameters can be roughly divided into 4 subcategories (Table_S_1: Overview of BFS assessment items sorted by subdomains of BFS):

I. physical performance [current training status of cardiovascular system, metabolism (endurance) and muscles (strength)] as well as the nutritional situation.
II. age-dependent sensory functions (hearing, vision, condition of the dental system).
III. current mental performance requirements in the psychomotor and cognitive areas.
IV. emotional and social areas.

The test battery for BFS assessment is a validated age- and sex-specific tool (objectivity 0.96, reliability 0.93, females age validity: total age correlation 85.2%; total age commonality in the main factor 76.3% [24]) (Table_S_2: Presentation of individual proceedings of BFS). The BFA is based on a sex-specific regression and factor analysis of functional age [21–24].

### BFA-index

For BFA-Index calculation the age of at least 35 is the model-required accepted cut-off value. BFA-Index is the resulting value of this mentioned regression model: BFA-Index = ∆CA–BFA (ye). It is specified in year-equivalents (ye) and is an important characteristic for BFS.

### Statistical methods

All data collected were analyzed using SPSS Statistics (version 24) for the present substudy. Various statistical tests were used for data processing: boxplots were used to identify outliers; Mann–Whitney *U* test, Kruskal–Wallis test, chi-square test, Spearman correlation, and ordinal logistic regression were used to analyze differences in location and correlations. The significance level was set at *p* < 0.05.

For the formation of three subject groups, threshold values for the range of CA-BFA were selected on the basis of the 1st (*n* = 88, CA-BFA > 13) and 3rd quartile (*n* = 85, CA-BFA < 3), therefore remaining *n* = 168 participants in the remaining subject group (CA-BFA 3 to 13).

First, the correlation of individual parameters with CA-BFA was examined in nonparametric correlation analysis. Significant correlations refer to possible candidates showing a rough correlation. Particular attention was then paid to those parameters for which a monotonic trend from functionally younger (1) to pre-aged (3) was observed and the differences between functionally younger (1) and age-appropriate (2) and between age-appropriate (2) and pre-aged (3) were significant.

Afterwards clinically relevant parameters that potentially might be influenced were selected and examined in more detail. In this way, 14 single parameters could be identified. Using a multiparametric regression model subsequently, some of the 14 parameters which as single parameters all showed a regression with *p* < 0.001, no longer showed any significance. Thus, the model was adjusted using the forward method, this resulted in 10 remaining parameters that seem to influence BFA.

## Results

### Characteristics of the cohort

Since the BFA can be calculated from the age of 35, in this study only participants over age of 35 were included. This collective (K35 +) consisted of 341 participants (50.1% of the original collective), including 269 females (78.9%) and 72 males (21.1%). Mean age was 51.2 ± 7.5 years (mean age for females 51.3 ± 7.6 years, for males 50.9 ± 7.3 years). BFA-Index was calculated for all subjects (Table 2: Age and BFA-Index for Subgroups). The most important results are described in the following.

**Table 2:** Age and BFA-index for subgroups

Population	Sub-group	*N*	Age		BFA-index		*d* (Age-BFA-Index)	
			Mean (SD)	Median (Q1; Q3)	Mean (SD)	Median (Q1; Q3)	Mean (SD)	Median (Q1; Q3)
Total	Total	341	51.7 (7.5)	52.0 (46.4; 57.7)	44.0 (8.6)	44.2 (38.1; 49.8)	7.7 (8.3)	8.1 (3.0; 13.3)
	Faster	85	46.7 (7.1)	47.1 (41.0; 51.8)	49.7 (8.3)	50.3 (42.9; 55.5)	− 3.0 (5.3)	− 1.1 (− 5.4; 0.7)
	Normal	168	52.3 (7.1)	53.0 (47.1; 58.3)	44.4 (7.4)	44.8 (39.8; 49.8)	7.9 (2.7)	8.1 (5.8; 10.1)
	Delayed	88	55.2 (5.9)	55.1 (50.8; 60.1)	37.6 (6.6)	37.7 (32.8; 43.0)	17.6 (3.7)	16.8 (14.6; 19.5)
Male	Total	72	51.4 (7.3)	50.9 (46–0; 56.6)	45.3 (7.9)	44.7 (40.7; 51.0)	6.1 (8.7)	7.2 (0.7; 12.5)
	Faster	23	45.8 (5.6)	45.6 (41.2; 49.6)	49.6 (7.6)	50.4 (43.1; 54.4)	− 3.8 (6.2)	− 0.9 (− 10.5; 0.6)
	Normal	32	53.3 (6.6)	53.7 (49.2; 58.8)	45.5 (6.5)	44.9 (42.8; 50.3)	7.8 (2.9)	7.5 (5.5; 9.9)
	Delayed	17	55.2 (6.5)	55.3 (50.5; 61.2)	38.9 (6.6)	40.6 (36.4; 43.8)	16.3 (3.1)	15.4 (14.1; 17.2)
Female	Total	269	51.8 (7.5)	52.1 (46.6; 56.0)	43.6 (8.7)	43.7 (37.4; 49.2)	8.1 (8.1)	8.4 (3.4; 13.3)
	Faster	62	47.1 (7.6)	48.1 (39.8; 52.2)	49.8 (8.5)	49.3 (42.3; 55.6)	− 2.7 (5.0)	− 1.2 (− 4.4; 0.7)
	Normal	136	52.1 (7.2)	52.6 (46.7; 58.3)	44.2 (7.6)	44.8 (39.0; 49.5)	8.0 (2.7)	8.2 (5.8; 10.1)
	Delayed	71	55.2 (5.9)	54.3 (50.8; 60.2)	37.3 (6.6)	37.0 (32.5; 43.0)	17.9 (3.8)	17.1 (15.1; 19.6)

*N* number, *SD* standard deviation, *Q* quartile *d* difference

### Influence of chronological age and gender

On average, the mean deviation of the difference between CA and BFA was 7.7 years (SD 8.3 years). Functionally normal ageing group showed a mean difference of 7.9 years (SD 2.7 years), functionally pre-ageing group a mean difference of − 3.0 years (SD 5.3 years) and the functionally delayed ageing group a mean difference of 17.6 years (SD 3.7 years).

The classification according to quantiles of the total provides comparable results for gender-specific analysis: 50.6% (*n* = 136) women aged functionally normal, 23.0% (*n* = 62) functionally pre-aged and 26.4% (*n* = 71) functionally delayed while 44.4% (*n* = 32) of men aged functionally normal, 31.9% (*n* = 23) functionally pre-aged and 23.6 (*n* = 17) functionally delayed.

Regarding differences between CA and BFA, Kruskal–Wallis Test confirmed statistical differences across all categories, mainly characterized by Chi^2^ test value (women Chi^2^ = 35,396; men Chi^2^ = 21,071, each with *p* < 0.001). Significant differences in both sexes were shown for a difference of CA to BFA in categories functionally normal to pre-ageing (women *Z* = − 4.115, *p* < 0.001; men *Z* = − 3.930, *p* < 0.001) as well as for women concerning functionally normal to delayed-ageing (*Z* = − 2.880, *p* = 0.004). No significant difference was shown for men functionally normal to delayed-ageing (*Z* = − 0.915, *p* = 0.367).

Equally, significant differences concerning functionally ageing were shown with regard to BFA-Index (Kruskal–Wallis-Test: women Chi-Quadrat 66,683; *p* < 0.001 and men Chi-Quadrat 17,864; *p* < 0.001). Concerning the different categories, there was a statistically significant difference in all groups: women functionally pre-aged to normal (*Z* = − 4.046; *p* < 0.001) as well as functionally normal to delayed ageing (*Z* = − 5.929, *p* < 0.001), men functionally pre-aged to normal (*Z* = − 1.979; *p* = 0.048) as well as functionally normal to delayed ageing (*Z* = − 3.130, *p* = 0.001).

### Potential confounding factors

No significant difference was shown using Kruskal–Wallis test for body mass index (BMI) (≤ 25 kg/m^2^ versus BMI > 25 kg/m^2^: men *p* = 0.434; women *p* = 0.194) although there is a tendency in women for lower BMI in functionally younger women (63.4% functionally younger, 57.4% functionally normal and 51.6% functionally pre-aged). The same applies for monthly income (*p* = 0.969) and for graduation (*p* = 0.554) although there was a trend for higher graduation in functionally younger participants.

Analyzed for alcohol consumption (up to one time a week versus more than one time a week) a significant difference (*p* = 0.008) was shown for functionally pre-aged drinking up to four times a month in 70.6%, functionally younger up to one time a week in 52.3%. Regarding physical activity to the point of sweating up to once a week versus more than once a week showed a significant difference (*p* < 0.001) in delayed ageing group with more physically activity [76.1% more than once a week versus 23.9% less than once a week as well as in functionally normal (73.2% versus 26.8%)]. Satisfaction with partnership showed a significant influence (*p* = 0.021) showing higher satisfaction over all age groups with higher satisfaction in the (functionally) younger (functionally delayed ageing group 83.0%, normal ageing group 74.3% and functionally pre-aged group 68.2%).

### Influencing factors

After carrying out the correlation analysis like described in chapter 2.5, clinically relevant parameters that potentially might be influenced were selected and examined in more detail. Using a multiparametric regression model subsequently, this resulted in an optimization value of *R*^2^ = 0.772. In this way, 14 single parameters could be identified:

Pulse performance index, Performance time (Subcategory I of BFS).

Start rate, Test motivation, Tapping basic rate (Subcategory II of BFS).

Cognitive reaction time, Verbal reaction time, Strategy building, Cognitive switching capability, Change-over capability, and Memory performance (Subcategory III of BFS).

Social potency, Sense of coherence, Complaint questionnaire (BFB total) (Subcategory IV of BFS).

Some parameters, which as single parameters all show a regression with *p* < 0.001, no longer showed any significance so that the model was adjusted using the forward method. After carrying out the regression analysis as well as cleanup by the forward method as described in Chapter 2.5, the following 10 parameters (*T* = 8.992; *p* < 0.001) remained as possible variables that can be influenced concerning biological ageing. These results are proofed by growing *R*^2^ from 0.253 up to 0.758 in the regression model (Table 3a: 14 BFS parameter, adjusted by age and gender and Table 3b: 8 BFS parameter, adjusted by age and Gender (forward method)). The remaining parameters are described in the following:

1. Pulse performance index (PPI) (*T* = − 11.546; *p* < 0.001): criterion for assessing the training status of the heart and circulation and metabolism. It serves as an indicator of the stress capacity and performance of the system (“cardio fitness”). Values below 1 are to be classified as poor. We find them in people who have a lack of exercise or exercise incorrectly. Values above 2 are considered good. Optimally endurance athletes can achieve values of 4. (Subcategory I of BFS).
2. Start Rate Tapping frequency part 1 (*T* = − 2.660; *p* = 0.008).
3. And Test motivation (Tapping frequency part 2) (*T* = − 3.326; *p* = 0.001): Start and preferred frequencies of the tapping test characterize psychomotor behavior and agility (Subcategory II).
4. Strategy building (*T* = 9.605; *p* < 0.001): parameter for assessing mental performance requirements in the cognitive area (Subcategory III).
5. Verbal reaction time (*T* = 7.144; *p* < 0.001): parameter for assessing mental performance requirements in the psychomotor and cognitive areas (Subcategory III).
6. Sense of coherence (SOC-L-9, *T* = − 2.719; *p* = 0.007): Coherence or self-assessed ability to cope with life (Subcategory IV).
7. Social power (*T* = 5.180; *p* < 0.001): The parameter essentially reflects the subjectively experienced stress caused by work and family (Subcategory IV).
8. Complaint questionnaire according to Höck and Hess (BFB total) (*T* = 3.659; *p* < 0.001): Index of well-being, indicates the subjectively perceived level of suffering and stress management techniques. (Subcategory IV).
9. Age (*T* = 8.999; *p* < 0.001).
10. Sex (*T* = − 8.143; *p* < 0.001).

**Table 3:** (**a**) 14 BFS parameter, adjusted by age and gender. (**b**) 8 BFS parameter, adjusted by age and gender (forward method)

(**a**) 14 BFS parameter, adjusted by age and gender						
BFS subdomain	Parameter	Unit	Coeff. B	Stand. beta	*T*	*p* value
I	Performance time	sec	− 0.033	− 0.026	− 0.771	0.441
I	Puls performance index	bpm	− 3.291	− 0.343	− 10.386	< 0.001
II	Motivation—part 2	Hz	− 1.033	− 0.077	− 1.536	0.125
II	Start rate—part 1	Hz	− 0.884	− 0.089	− 2.504	0.013
II	Tapping—part 3	Hz	− 0.454	− 0.043	− 0.946	0.345
III	Cognitive reaction time	sec	0.186	0.051	1.156	0.249
III	Cognitive switching capability	sec	0.046	0.048	1.364	0.173
III	Memory performance	n	0.034	0.104	1.622	0.106
III	Orientation capability	n	1.407	0.078	0.757	0.449
III	Strategic thinking	sec	0.019	0.176	1.566	0.118
III	Verbal reaction time	sec	0.792	0.177	4.215	< 0.001
IV	Social dominance (SOC-L9)		− 0.101	− 0.084	− 2.670	0.008
IV	Social potence		0.263	0.162	5.515	< 0.001
IV	Wellbeing total		0.166	0.121	3.494	0.001
	Age	years	0.311	0.273	9.036	< 0.001
	Gender	f/m	− 4.802	− 0.208	− 7.916	< 0.001

(b) 8 BFS parameter, adjusted by age and gender (forward method)						
BFS subdomain	Parameter	Unit	Coeff. B	Stand. beta	*T*	*p* value
I	Puls performance index	bpm	− 3.261	− 0.340	− 11.546	< 0.001
II	Motivation—part 2	Hz	− 1.542	− 0.115	− 3.326	0.001
II	Start rate—part 1	Hz	− 0.931	− 0.084	− 2.660	0.008
III	Strategic thinking	sec	0.032	0.299	9.605	< 0.001
III	Verbal reaction time	sec	0.959	0.214	7.144	< 0.001
IV	Social dominance (SOC-L9)		− 0.102	− 0.095	− 2.719	0.007
IV	Social potence		0.246	0.152	5.180	< 0.001
IV	Wellbeing total		0.172	0.126	3.659	< 0.001
	Age	years	0.307	0.269	8.999	< 0.001
	Gender	f/m	− 4.927	− 0.234	− 8.143	< 0.001

*BFS* Biofunctional Status, *Coeff. B* Coefficient Beta, *Stand. Beta* Standardized Beta, *T* *T* value, *bpm* beats per minute, *HZ* Herz, *sec* seconds, *n* number, *f/m* female/ male

Of the ten identified parameters, one parameter can be assigned to subcategory I of BFS (PPI). Two parameters can be allocated to subcategory II (Tapping frequency part I and II), two parameters to subcategory III (strategy building and verbal reaction time) and three parameters to subcategory IV (SOC-L-9, social potency, BFB total). Age and sex are no subcategories of BFS, nevertheless, they have an influence on BFA and BFA-Index.

## Discussion

While CA cannot be influenced, we could show that in contrast, bio-psychosocially ageing can be influenced by several factors. The most promising approach to this is, according to our results, to support low social stress, high social integration and a good sense of coherence as well as maintaining a good mental and cognitive status. Physical and cognitive factors, on the other hand, tended to correlate with CA rather than BFA. Bio-functionally delayed ageing participants achieved better values in physical or cognitive parameters than bio-functionally prematurely aged subjects. However, the differences between the bio-functional age groups were more often statistically significant in the mental performance and emotional-social subcategory (subcategories III and IV of BFS).

PPI is used to assess the training status of the heart and circulation as well as the metabolism. It is an indicator of the stress ability and performance of the system (cardio fitness). Values below 1 are to be classified as poor and can be found in people who have a lack of exercise or exercise incorrectly. Values above 2 are considered good. Optimally training endurance athletes can reach values of 4 [24]. PPI can be influenced by regular and correct physical activity and thus may contribute to the influence of well-maintained vitality.

Start rate and test motivation as part of the tapping test, provide information about psychomotor endurance (checking the basic psychomotor speed) and about functional maturity (characterized by psychomotor behaviour and agility) [25, 26]. The influence of psychomotor activity on quality of life as well as the influenceability through habitual physical activity has already been proven individually [27, 28].

Strategic thinking as part of the labyrinth test indicates problem solving behaviour as a part of memory performance verified by this test. The underlying principle is based on the standardized evaluation of error and time values. It is known that all four dimensions examined in this test (orientation ability, planning ability, problem-solving behaviour and conversion ability) decline with increasing age. In particular, the ability to concentrate to perform this test procedure decreases while ageing [30]. Thus, we are the first to explore that strategic thinking and underlying concentration obviously have an influenceable part regardless of age. It has to be proven that vitality might be influenced by concentration training.

Verbal reaction time as another parameter for mental performance also seems to be influenceable. In literature, intraindividual variability of reaction time has been studied in several investigations in connection with mortality and dementia. As far as we know, no investigations concerning verbal reaction time have been made so far, therefore we may assume that mental and concentration training help to maintain vitality.

Sense of coherence (SOC_L_9) is based on the concept of salutogenesis by Antonovsky, e.g. comprehensibility (belief that things happen in a predictable fashion), manageability (believe that the resources necessary to take care of things are available), and meaningfulness (believe that things in life are a source of satisfaction) [29]. The original questionnaire developed by Antonovsky was adapted to and validated in German [30, 31]. The two remaining subjective parameters physical and emotional wellbeing reflect subjective assessments of subjects about their state of health and have been proven to be dependent on biological age. The underlying questionnaire is based on WHO criteria and distinguishes subjects with functional neurotic disorders from so-called normal mental subjects [24]. Thus, “normal” mental health seems to be a desirable status from the perspective of healthy ageing.

The “Giessen Test” examines various dimensions of social competence. Social power as one of them refers to emotional adaptability. It is already known from the literature that social competencies improve with increasing age due to longer experience in a professional or private environment [7], therefore, our results proof the concept of experience.

BFS total as an index of well-being, indicates the subjectively perceived level of suffering and stress management techniques. Certain complaints lead to a measurable reduction in vitality. These physical and psychological symptoms usually have a kind of valve function for disorders whose causes lie in completely different areas (e.g. incorrect physical demands or social stress). Mood disorders can not only indicate physical underload or psychosocial stress but also how these experiences are processed. Physical and emotional wellbeing reflect subjective assessments of subjects about their state of health and have been proven to be dependent on biological age. The underlying questionnaire is based on WHO criteria and distinguishes subjects with functional neurotic disorders from so-called normal mental subjects [24].

Age and Sex as two discovered influencing parameters on BFA are not part of the BFS, but nevertheless they obviously influence BFS and therefore BFA and vitality. Chronological age may not be changed. Since no significant difference was shown for men functionally normal to delayed-ageing (*Z* = − 0.915, *p* = 0.367) we assume that this could be explained by the fact that the career advancement phase, including family, is more strenuous than the later phase before the end of the career, in which an increasing serenity of everyday life deploys.

Whether gender or to be more precise sexual hormones and hormone replacement therapy might influence vitality apart from biological gender could be a future interesting question of studies (e.g. participants in transgender therapy may take part).

The inclusion of subjects aged 35–65 years is one of the strengths of this study. Due to the results, a statement about delaying the ageing process can be made. In the present study, the velocity of the ageing process was the end point. This is in contrast to other studies, which have studied in particular people from older adulthood and explored relationships between bio-psychosocial factors and mortality. Thus, the advantage is seen in the fact, that in line with the concept of active ageing, bio-psychosocially successful ageing, vitality and well-being seem to be desirable and not merely delaying the end of life.

The test for various confounders showed that the regression model is valid since BMI, monthly income and graduation do not show any influence. Although physical activity showed an influence, we assume this to be explainable because physical condition as an influencing factor on the ageing process have already been proven. This is also supported by the finding of this investigation since parameters that measure physical performance like PPI showed a positive result. As well, higher satisfaction with partnership the (functionally) younger participants felt, may support the assumption that emotionally stressful environments contribute to a faster functional ageing process (supported by the fact that the sense of coherence and BFBS total showed a positive result).

People in managerial positions and with high incomes tend to be younger. It is a social phenomenon that alcohol fits in with a “successful appearance”. It turns out that people in management positions in particular drink alcohol more frequently. Frequency of alcohol is related to successful professional life (management/income), professional life is related to the BFA so the frequency of alcohol also appears to be related to the BFA-Index, therefore we assume a spurious correlation.

One weakness of the present study is the low case rate (*n* = 341), especially for male subjects (*n* = 88), associated with low significance of the statistical calculations. In particular, a division into age groups was therefore not reasonably possible. Therefore, to exaggerate, results of 35-year-old participants have been compared to those of 65-year-old participants. Another weakness could be that the test subjects were not screened for mental illnesses such as depression or the like.

Since the subjects were able to volunteer for the study and, apart from an evaluation of the personal BFS status, no reward was offered for participation, a greater number of healthy and health-conscious or interested individuals may have volunteered. It is therefore conceivable that the results of the study would have been slightly different in the case of a collective that corresponded more to the average population.

According to WHO the ageing population is the most important medical and social demographic problem worldwide. Therefore the WHO has defined healthy ageing as a process of maintaining functional ability to enable wellbeing in older age [32]. In view of these facts—as well as mentioned at the outset—population growth and increasing life expectancy, the question arises as to what extent politics, society and every individual can contribute to maintaining the quality of life of every person into old age and enabling social and economic integration. The concept of active ageing involves promoting a holistic view of the ageing process with its biological as well as cognitive-mental, psychological, emotional and social components. For some years now, programs and action strategies have been developed at a social, economic and political level with the aim of promoting active ageing in order to tap into and maintain the potential of the ageing population [3].

The determination of bio-functional age (BFA) allows the recording of numerous physical, cognitive, emotional and social components and thus enables a more precise assessment of a person’s current functional state than it is possible on the basis of chronological age. The BFA survey offers an opportunity to map a person’s vitality and well-being and to track them over time through repeated measurements [7, 8]. By knowing the individual’s biopsychosocial functional state, the individual is enabled to improve vitality and quality of life by using existing and promoting missing biopsychosocial strengths and resources.

## Supplementary Information

Below is the link to the electronic supplementary material.

Supplementary file1 (DOCX 21 KB)

Supplementary file2 (DOCX 19 KB)

## Data Availability

Data may be available on request.
